# Design of a WSN for the Sampling of Environmental Variability in Complex Terrain

**DOI:** 10.3390/s141121826

**Published:** 2014-11-18

**Authors:** Miguel A. Martín-Tardío, Ángel M. Felicísimo

**Affiliations:** Centro Universitario de Mérida, Universidad de Extremadura, Mérida 06800, Spain; E-Mail: amfeli@unex.es

**Keywords:** wireless sensor network, geographic information systems, binary integer programming, environmental monitoring, viewshed analysis, network optimization

## Abstract

*In-situ* environmental parameter measurements using sensor systems connected to a wireless network have become widespread, but the problem of monitoring large and mountainous areas by means of a wireless sensor network (WSN) is not well resolved. The main reasons for this are: (1) the environmental variability distribution is unknown in the field; (2) without this knowledge, a huge number of sensors would be necessary to ensure the complete coverage of the environmental variability and (3) WSN design requirements, for example, effective connectivity (intervisibility), limiting distances and controlled redundancy, are usually solved by trial and error. Using temperature as the target environmental variable, we propose: (1) a method to determine the homogeneous environmental classes to be sampled using the digital elevation model (DEM) and geometric simulations and (2) a procedure to determine an effective WSN design in complex terrain in terms of the number of sensors, redundancy, cost and spatial distribution. The proposed methodology, based on geographic information systems and binary integer programming can be easily adapted to a wide range of applications that need exhaustive and continuous environmental monitoring with high spatial resolution. The results show that the WSN design is perfectly suited to the topography and the technical specifications of the sensors, and provides a complete coverage of the environmental variability in terms of Sun exposure. However these results still need be validated in the field and the proposed procedure must be refined.

## 1. Introduction

Climate mapping is required for numerous environmental studies, but methods based on weather stations have limitations in terms of low spatial resolution and unsuitable spatial distribution of the weather stations. The common procedure interpolates the data using splines, kriging or other methods [1–5]. A well-known climatic dataset is Worldclim, elaborated from thousands of stations [6] and widely used, for example, to provide the independent variables in predictive ecological modelling [7,8].

The real spatial resolution of climatic datasets is on the order of 5–25 km or more. This makes them useful only for coarse scale studies. However, many studies require spatial resolutions of tens of metres or less; for example, if the habitat of animals or plants closely linked to microclimate conditions must be modelled. The problem is very important in mountainous areas, where spatial heterogeneity is very high due to the different exposures to solar radiation and topographic shadows.

One of the ways to complete climate data is to place temperature and radiation sensors on the ground. In this context, a wireless sensor network (WSN) is a self-organized wireless communication network composed of a large number of sensor nodes interacting with the physical world [9].

In recent years, *in-situ* environmental parameter measurements using sensor systems connected to a wireless network have become widespread [10–34]. This increased interest in the application of WSNs for environmental science research has already been highlighted in [12], but the problem of monitoring large and topographically complex (mountainous) areas by means of WSNs is not well resolved.

There are several reasons for this: (1) the environmental variability is unknown in the field; (2) without this knowledge, a huge number of sensors would be necessary to ensure complete coverage of the environmental variability and (3) WSN design requirements, for example, effective connectivity (intervisibility), limiting distances and redundancy, are usually solved by trial and error.

Using temperature as the target environmental variable, we propose: (1) a method of determining the homogeneous environmental classes to be sampled using the digital elevation model (DEM) and geometric simulations and (2) a method to determine an effective WSN design in complex terrain in terms of the number of sensors, redundancy, cost and spatial distribution. The proposed methodology can be easily adapted to a wide range of applications that need environmental monitoring.

Therefore, the specific objectives are to define of homogeneous classes to be sampled, to select the candidate area for each class with sensor and to optimize network design based on the physical constraints that guarantee coverage of environmental variability.

## 2. Some Considerations on the Design of an Environmental WSN

From the literature review we found that in complex terrains WSNs are deployed intuitively without specific analysis of the location of each sensor node. Since WSNs work best with the nodes organized and interconnected in a hierarchical clustering schema [35–37], the design of a WSN must take into account at least two criteria: (1) the role of the nodes in the network (network architecture) and (2) the diffusion characteristics of wireless signals between nodes (coverage and interconnectivity) [38,39].

**Figure 1.**
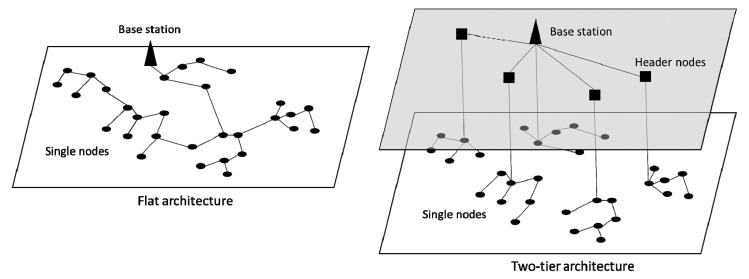
Flat architecture *vs.* two-tier architecture.

Regarding network architecture, we propose a hierarchical two-level tree topology (two-tier architecture, Figure 1). The first level consists of single nodes (SNs) grouped into clusters where each SN is able to communicate directly or indirectly and with a unique header node (HN). Communications are established over short or moderate distances.

At the second level, the HNs must communicate with a base station (BS) that can be one of them or a complementary device. The distances can be significantly longer in this case and it may be required that the nodes are able to communicate with the BS through radio links or mobile telephony networks (GSM/GPRS). Finally, the BS serves to periodically send all information of the nodes to a remote processing system, also via radio or GSM/GPRS. The optimization of the WSN includes two objectives:

(1) to use only the necessary nodes (both SN and HN) and
(2) to ensure coverage/sampling of the environmental variability.

Obviously, fewer nodes involve lower cost, energy saving and easier maintenance [9]. Using a two-tier architecture helps to reduce the number of nodes involved in data transmission over long distances with a BS [35].

## 3. Material and Data

### 3.1. Digital Elevation Model (DEM)

**Figure 2.**
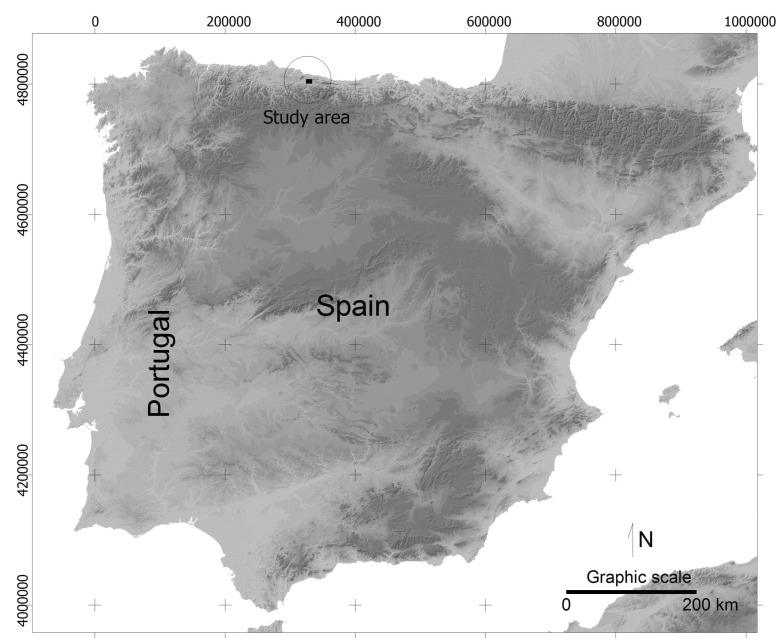
Study area in northern Spain (Asturias).

**Figure 3.**
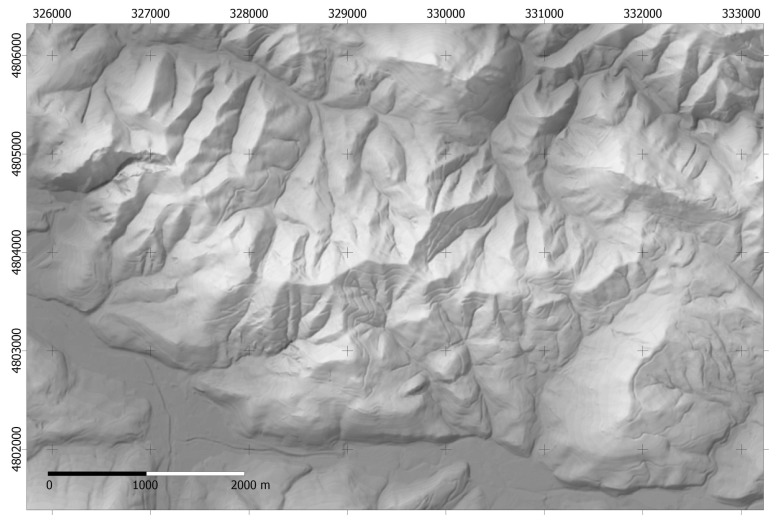
Hill-shaded DEM of the study area (9645 × 6690 m, 5 m spatial resolution).

The proposed methodology has been applied to a mountainous area in northern Spain with dimensions 9645 × 6690 m (Figures 2 and 3) and pixel size of 5 m. Elevation is in the range 15–750 m. The DEM proceeds from LIDAR data and the estimated accuracy is better than ±1 m. The lower left corner of the area has XY coordinates 324,400 and 4,800,500 (Datum WGS, projection UTM, zone 30N).

### 3.2. Software

The geographic information systems (GISs) ArcInfo and ArcView (ESRI Inc., Redlands, CA, USA) have been used for map analysis, including the tasks of modelling the solar irradiance, terrain classification, intervisibility analysis and representative areas selection. The LINGO tool [40] has been used to build and solve optimization models based on integer linear programming (ILP) that determines a suitable set of candidate nodes satisfying the design objectives.

## 4. Methods

The proposed methodology is carried out in four steps: (1) modelling the potential irradiance; (2) defining and mapping homogeneous classes; (3) selecting the network nodes and (4) optimizing the network topology.

### 4.1. Modelling the Potential Irradiance

The main variable driving the surface temperature of a topographically complex area is solar radiation. The current temperature maps may include the influence of elevation using estimated temperature gradients, but they do not consider the angle of exposure to solar beams and the topographic hill-shade. Consequently, the local variations due to relief are lost. The relief influence can be estimated by means of simulating the Sun's trajectory and its incidence angle over each cell in the DEM [41]. For this objective we have used Zimmermann's shortwavc.aml routine [42]. The routine uses the latitude of the study zone and the solar declination to calculate the Sun's azimuth and zenith angles for given hourly values. Daily values are estimated by integrating the hourly value intervals and local shadows are taken into account if they occur.

In this work, solar radiation has been estimated for the average day of each month. The result is a set of 12 monthly irradiance models (kJ·m^−2^·day^−1^) that represent the variation in solar radiation exposure throughout the year with a 5 m spatial resolution.

### 4.2. Mapping Homogeneous Classes

With elevation and solar radiation being the main causes of local variations in environmental conditions, we undertook an analysis to define and delimit a set of homogeneous classes, taking into account all solar radiation maps and the DEM.

The method uses the iterative self-organizing data analysis technique algorithm (ISODATA) [43] widely used in the unsupervised classification of satellite images, terrain landforms and pattern recognition. ISODATA is a well-known algorithm based on an iterative procedure that reclassifies the pixels (cells in this case) until an optimal classification of a predefined number of classes is achieved. As result, the terrain is tessellated in areas with maximum internal (intra-area) homogeneity and maximum external (inter-area) heterogeneity covering all the solar radiation variation in the study area.

We used the 12 solar radiation maps to perform the ISODATA classification. The only parameter to be defined was the number of classes to be separated by the algorithm. Since we intend to conduct a field trial in the future, we chose a total of 20 classes to keep the number of sensors within a reasonable limit, but this number can be changed depending on available resources. As expected, a lot of areas his assigned to each class (more than 25 in all the classes). The following step is to select only a sample of these areas as candidates to contain a sensor node.

### 4.3. Preselecting Candidate Areas and Nodes within Classes

The result of the previous step is a map of polygons where each element is assigned to a class from 1 to 20. Usually, each class has many polygon areas that can be used as candidate areas to carry a sensor. The areas have been filtered by selecting the 20 larger polygon areas for each class. This criterion is used to reduce the magnitude of the subsequent analysis but also to select areas with significant extensions. Smaller areas are discarded to facilitate the placement of sensor nodes in the field avoiding errors. The result is a set of approximately 400 candidate polygons to represent the 20 homogeneous classes. As the potential location of a sensor, a node is placed automatically in the centroid of each polygon.

### 4.4. Testing Connectivity

Two nodes cannot connect to each other if the terrain breaks the line of sight (LOS). Therefore, each LOS between each pair of nodes must be “labelled” with a value of intervisibility: true or false. This analysis was undertaken with standard GIS tools taking into account not only the relief but also the limiting angles, both horizontal and vertical, and distance. The values of these factors depend on the characteristics of the selected model. We chose a device whose characteristics are shown in Table 1.

Since the antennas are omnidirectional, a valid horizontal angle includes the full range 0–360*°* but the vertical angle is restricted to ±15*°* relative to the horizontal. The maximum connectivity distance included in the visibility analysis is 5000 m. Distance has a strong influence on energy expenditure and, therefore, on the lifetime of the network [9]. Finally, two statistics are calculated for each node: the number of intervisibility connections and the mean distance to visible neighbours.

The analysis taking into account the node location, relief, valid angles and maximum distance generates a 400 × 400 symmetric matrix of intervisibility with values 0 (not visible) or 1 (visible). This matrix restricts the paths of connectivity and consequently the potential network design.

**Figure 4.**
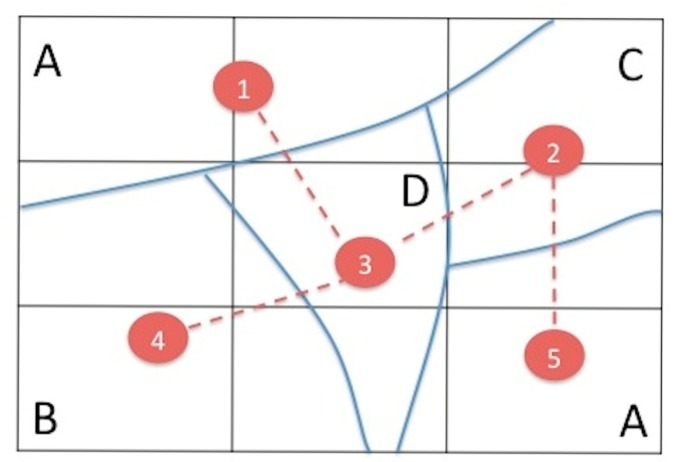
A minimalist example of five nodes and four classes.

Figure 4 shows a minimalist example to clarify the procedure. The study area has been segmented into five zones (nodes numbered 1 to 5) representing four homogeneous classes (A, B, C, D). Class A has two candidate zones (1 and 5). Solid lines separate the zones and dotted lines represent the intervisibility among the nodes. Node pairs 1–2, 1–4, 1–5, 2–4, 3–5, and 4–5 are not intervisible.

Table 2 shows the matrix of relationships among the nodes. The values 0–1 represent the condition of intervisibility between nodes and the columns VN (visible nodes) and MD (mean distance) represent the number of VNs including itself, and the MD to the visible neighbours. X and Y are the coordinates of each node. HC represent the homogeneous class that the node belongs.

### 4.5. Selecting the Nodes

The next step is to select the best subset of nodes from the potential candidates that configures a functional network with or without redundancy. There are many examples of optimization algorithms based on clustering and node location [37,44] but they focus on the optimization of network architecture from device features and the limitations of hardware and software. It is less common to find examples of optimization based on the representativeness of environmental conditions [22,38,45].

We used some single rules to select the best functional configuration from the connectivity matrix:

(i) From all the candidates for each class the node with the highest number of intervisibility connections with its neighbours will be chosen.
(ii) If two or more nodes tied, the one with the lowest average distance to the visible neighbours will be chosen.

The result of applying the criteria (i) and (ii) to the example is a reduction in the number of candidate nodes. Node 1 and node 5 are placed in representative zones of the same HC and both nodes have the same number of neighbours. However, node 5 is discarded because of the higher mean distance to its neighbours compared to node 1. The following step determines which of the selected sensor nodes also acquires the HN role.

In the example we selected a unique node to represent each HC. However, this design is more vulnerable than a network with more representative zones for each HC. The same criteria used up to now can be applied to select two or more nodes for each HC. Redundancy is more expensive in terms of material and administration but increases the availability, security and fault tolerance of the network. We believe that a minimum of two nodes for each HC should be selected.

### 4.6. Optimizing the Network Topology

Research related to node placements in WSNs is growing. The main proposed methodologies exposed in [37,38,46] focus on this problem as multi-objective optimization. The two most common approaches are: (1) a pure multi-objective metaheuristic methodology mainly based on genetic [46–49] and evolutionary [50,51] algorithms; and (2) an aggregate multi-objective methodology based on linear programming optimization models (LP) [35,52–56]. We use the LP approach.

According to network design considerations (Section 2), the optimization process should cluster the nodes to determine which also acquire the HN role. The goal is to minimize the number of HNs, maintaining full network connectivity and functionality.

The optimization problem has been approached by means of binary integer programming (BIP) as: (1) the mathematical optimization model approaches the known “set covering problem” [57] of operations research; (2) the model, based on an objective function, variables and constraints, provides simplicity and ease of understanding and (3) software tools are available that simplify the creation of these models for application of these models to large data matrices.

To establish a model based on BIP, the first step is to declare the variables:

$$\begin{array}{c} Xx,y\in \left\{0,1\right\}\\ Xx,y=1,HN\text{is placed in position}x,y\\ Xx,y=0,\text{otherwise} \end{array}$$

Second, the objective function must be defined:

$$\min Z=\sum_{x,y}Xx,y$$

Finally, the constraints of the system are based on the conditions:

1. Any network node can become an HN.
2. Two network nodes are connected if there is a relationship of intervisibility between them.
3. The sum of candidate nodes around any node must be equal to or greater than 1, if not this node will be considered as an HN.

Once the model is developed, finding an optimal solution can be undertaken by the simplex or branch-and-bound algorithm (B-and-B). Although the simplex algorithm is the general method for LP, the B-and-B algorithm is suitable when working with binary variables [40,58].

The procedure applied to the above example using node ID instead of the (x, y) coordinates begins with the declaration of the variables Xi:

$$\begin{array}{c} Xi\in \left\{0,1\right\}\\ Xi=1,HN\text{is placed in position}x,y\\ Xi=0,\text{otherwise} \end{array}$$

In the constraints definition, it is necessary to obtain the candidate nodes resulting from the previous phase (see Table 2). For example, to ensure that the node with ID = 1 meets the conditions defined above, it is included as a constraint:

X1+X3≥1

Adding constraints for all the candidate nodes and the objective function, the proposed model is:

$$\begin{array}{c} \min Z=\underset{i}{\text{∑}}Xi,\text{where}Xi=\left\{0,1\right\}\forall i=1..4\\ s.t.\\ X1+X3\geq 1\left(ID\;\text{node}=1\right)\\ X2+X3\geq 1(ID\;\text{node}=2)\\ X1+X2+X3\geq 1\left(ID\;\text{node}=3\right)\\ X3+X4\geq 1\left(ID\;\text{node}=4\right) \end{array}$$

**Figure 5.**
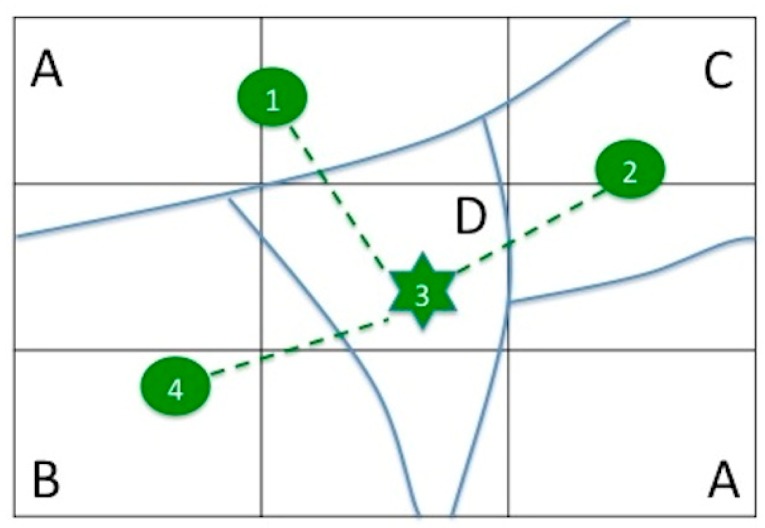
Optimal network design proposed with node ID = 3 as HN.

The theoretical model will be implemented as an optimization model using the LINGO software tool (optimization modelling software for linear, nonlinear and integer programming) that can read data from external matrices and find an optimal solution regardless of the number of nodes available. This software automatically recognizes this model as a BIP model and applies the B-and-B algorithm to find the optimal solution. Figure 5 shows the proposed network design from the solution obtained. This solution determines a unique HN corresponding to the node ID = 3 (star symbol).

## 5. Results

The proposed methodology has been applied to the study area described in Section 3.

### 5.1. Modelling the Potential Irradiance

**Figure 6.**
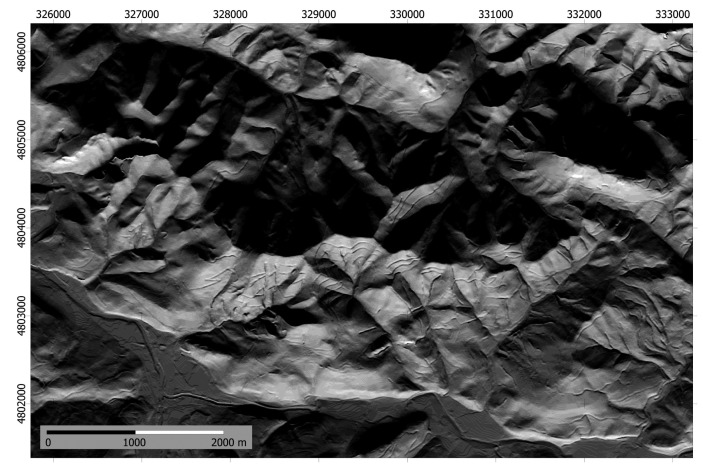
Potential solar radiation corresponding to day 349 (15 December each year); the values are in the range from 0 (black, without direct solar radiation) to 16,000 kJ·m^−2^·day^−1^ (white).

The result of this stage is a set of 12 monthly irradiance models (kJ·m^−2^·day^−1^) with 5 m spatial resolution that represent the variation in solar radiation exposure throughout the year. As an example, Figure 6 shows the model corresponding to day 349 (15 December each year), near to the winter solstice.

### 5.2. Mapping the Homogeneous Classes

**Figure 7.**
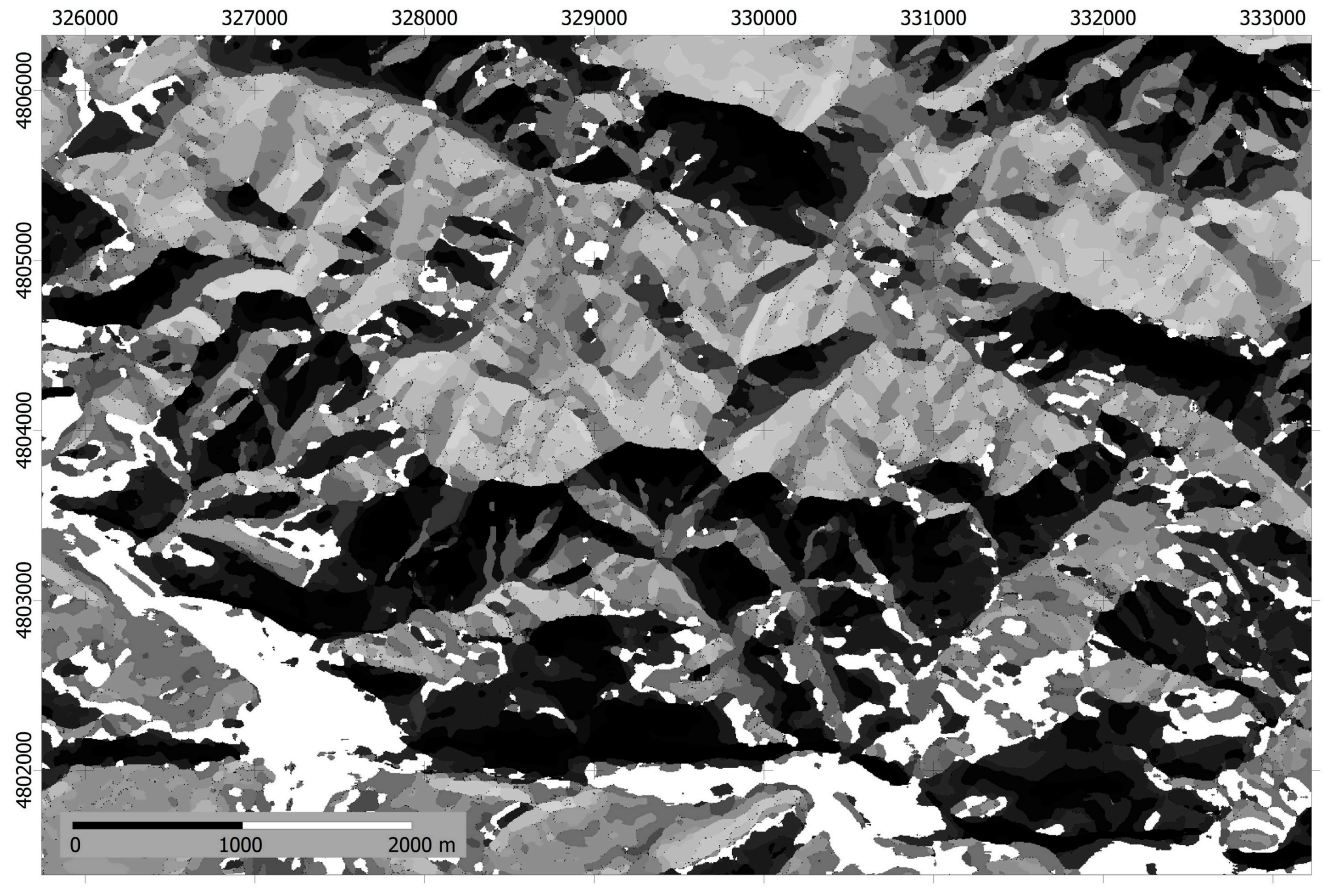
Homogeneous classes symbolized with grey tones. As expected, the appearance is similar to the irradiance spatial patterns. For clarity, class 14 has been represented as white areas.

The result of applying the ISODATA to the stack of elevation and irradiance models is terrain segmentation into 20 homogeneous environmental classes. Figure 7 shows the appearance of the areas symbolized in grey tones. Class 14 is represented as white areas.

### 5.3. Preselecting Candidate Nodes

**Figure 8.**
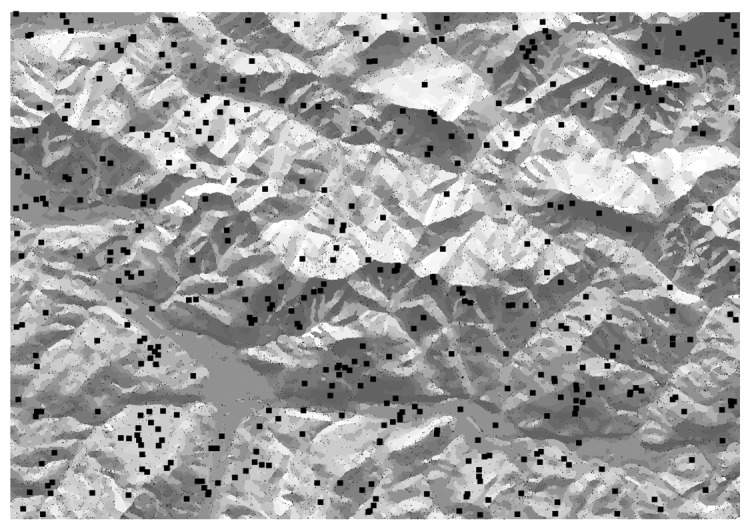
425 preselected candidate nodes (around 20 for each class).

The number of representative areas of each class is usually very high but mostly contains very few cells. As explained before, around 20 large areas of each class have been selected as candidate areas, discarding the rest. If the distance and visibility analysis generates few candidate areas, it will likely be necessary to change the characteristics of the sensors to increase the distance connectivity. Figure 8 shows the preselected 425 candidate nodes as black points.

### 5.4. Testing Connectivity (Intervisibility) and Distance

The intervisibility has been calculated for each node pair including the restrictions of vertical angles (±15°) and maximum distance (5000 m). A set of more than 112,000 visibility tests were performed automatically by a GIS and the results have been used to build the 425 × 425 intervisibility matrix. Complementary columns with the coordinates, the number of VNs and the MD to them have been added as an example in Table 2.

**Figure 9.**
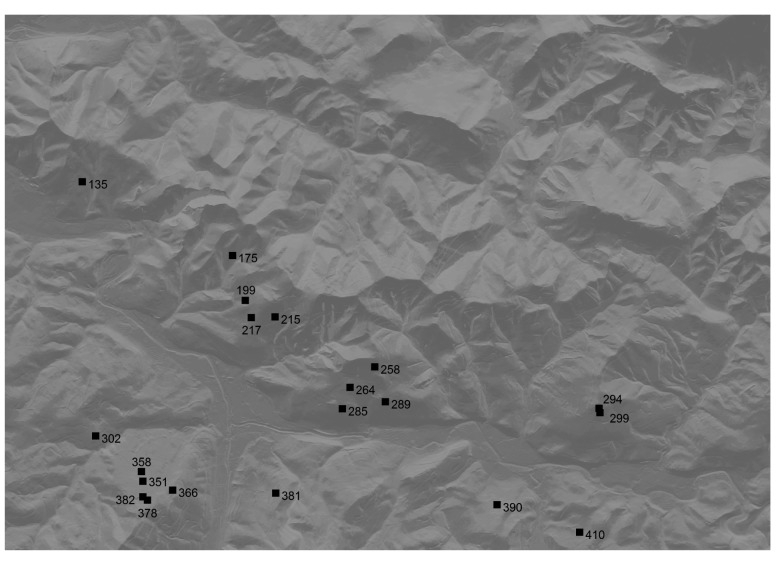
Selected nodes based on the maximum intervisibility and minimum distance to neighbours.

The procedure described in Section 4.5 has been applied and a set of 20 network nodes based on the maximum intervisibility and minimum distance to other nodes has been selected, one for each class (Figure 9).

### 5.5. Optimizing the Network Topology

**Figure 10.**
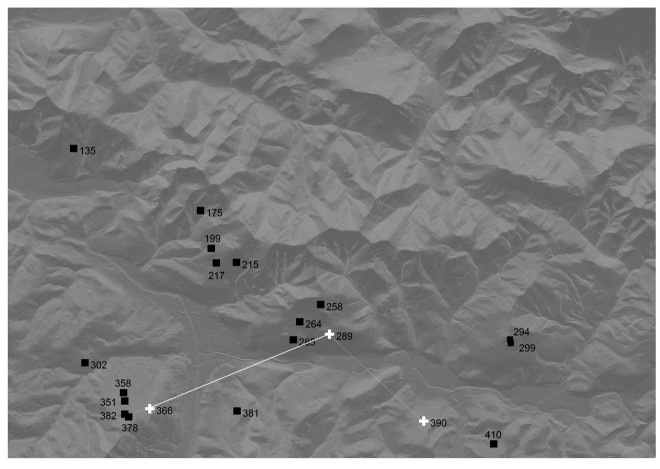
Optimized WSN with three HNs (white crosses). White lines are the intervisibility between HNs. Node 289 is the most appropriate to be configured as the BS.

The process carried out by means of the BIP mathematical model generates the optimal topology. Figure 10 represents the deployment of the WSN of 20 nodes (without redundancy) where the HNs are represented as white crosses. Nodes 289, 366, and 390 have been selected as HNs and, because the selection is based on distance and connectivity, almost all sensor nodes are connected simultaneously with two HNs so increasing the reliability and fault tolerance.

The HNs are interconnected as: 366 ↔ 289 ↔ 390, 366 and 390 remaining isolated from each other. This circumstance suggests the use of node 289 as the BS (see Section 2). The BS node is responsible for communicating via radio links or telephony (GSM/GPRS), sending all the information periodically to a remote system for processing.

We emphasize that a second network can be added for redundancy. In this case, a new set of HNs is defined and the interconnectivity should be analysed to define the optimum BS for long distance communication.

## 6. Conclusions and Future Work

The results show that the proposed methodology can be used for optimizing the design of a WSN that meets the objectives of this work:

1. A design adapted specifically to the study area. The network design is perfectly suited to the topography and the technical specifications of the sensors.
2. Complete coverage of the environmental variability in terms of Sun exposure, the main factor for local contrast on a detailed scale.
3. An adaptable network from the minimalist version with a unique node for each class to the progressive redundant networks for greater fail tolerance.
4. A robust topology based on intervisibility analysis that takes into account the technical properties of the sensors.

The methodology has some other features that allow greater flexibility. For example, it is possible to use sensors with different ranges and dispersion angles because these properties are fully configurable for each node in the visibility analysis. Likewise, once the network is configured, the distances between the nodes can be revised and the sensor model modified to reduce the cost using cheaper devices.

The proposed procedure, although functional, must be refined. For example, the selection of candidate nodes can be carried out with more flexibility using some degree of randomization. Similarly, the ISODATA classification used to define the classes to be sampled can be revised to assign greater weight to elevation, a factor with a strong influence on temperature values.

Further work includes the ground validation of this methodology. Sun exposure is the main factor driving the microclimate variability in complex terrain but the percentage of explained variability and the accuracy of the system are unknown. In this context, it is necessary to deploy a real network over a control terrain. Currently, we are developing the physical implementation of the sensors based on open hardware devices.

Since thermal infrared emissivity is strongly related to surface temperature, further work also includes the use of the thermal band of Landsat satellites to compare and analyse the sensor data. We think that the proposed networks and satellite thermal imagery can help in building high-resolution temperature maps based on geometrical models and real terrain data. As noted at the beginning of this paper, many environmental studies require spatial resolution of tens of metres, especially for species conservation closely linked to microclimatic conditions.

## Figures and Tables

**Table 1.** Summary of the technical features of sensor nodes and header nodes.

**Protocol**	**IEEE802.15.4/ZigBee-Pro**
Antenna	Dipole 5 dBi
Frequency	2.40–2.48 GHz
Power TX	50 mW
Max. distance	5000 m
Horizontal angle	0–360°
Vertical angle	±15°

**Table 2.** Connectivity matrix for the example in Figure 4 (arbitrary units).

**HC**	**Nodes**	**X**	**Y**	**1**	**2**	**3**	**4**	**5**	**VN**	**MD**
A	1	1.1	2.5	1	0	1	0	0	2	1.1
B	2	2.5	2.0	0	1	1	0	1	3	1.2
C	3	1.6	1.3	1	1	1	1	0	4	1.1
D	4	0.7	0.8	0	0	1	1	0	2	1.0
A	5	2.5	0.7	0	1	0	0	1	2	1.3

## References

[1] Hutchinson M.F. (1991). The application of thin plate smoothing splines to continentwide data assimilation. BMRC Res. Rep..

[2] Hutchinson M.F. (1991). Climatic analyses in data sparse regions. Climatic Risk in Crop Production: Models and Management for the Semiarid Tropics and Subtropics.

[3] Collins F.C., Bolstad P.V. A comparison of spatial interpolation techniques in temperature estimation.

[4] Saveliev A.A., Mucharamova S.S., Piliugin G.A. (1998). Modeling of the daily rainfall values using surfaces under tension and kriging. J. Geogr. Inf. Decis. Anal..

[5] Ninyerola M., Pons X., Roure J.M. (2000). A methodological approach of climatological modelling od air temperature and precipitation through GIS techniques. Int. J. Climatol..

[6] Hijmans R.J., Cameron S.E., Parra J.L., Jones P.G., Jarvis A. (2005). Very high resolution interpolated climate surfaces for global land areas. Int. J. Climatol..

[7] Bedia J., Herrera S., Gutiérrez J.M. (2013). Dangers of using global bioclimatic datasets for ecological niche modeling. Limitations for future climate projections. Glob. Planet. Chang..

[8] Mateo R.G., de la Estrella M., Felicísimo Á.M., Muñoz J., Guisan A. (2013). A new spin on a compositionalist predictive modelling framework for conservation planning: A tropical case study in Ecuador. Biol. Conserv..

[9] Akyildiz I.F., Su W., Sankarasubramaniam Y., Cayirci E. (2002). Wireless sensor networks: A survey. Comput. Netw..

[10] Oliveira L.M., Rodrigues J.J. (2011). Wireless Sensor Networks: A Survey on Environmental Monitoring. J. Commun..

[11] Hart J.K., Martinez K. (2006). Environmental sensor networks: A revolution in the earth system science?. Earth-Sci. Rev..

[12] Porter J., Arzberger P., Braun H.-W., Bryant P., Gage S., Hansen T. (2005). Wireless Sensor Networks for Ecology. Bioscience.

[13] Polastre J., Szewczyk R., Mainwaring A., Culler D., Anderson J., Raghavendra C.S., Sivalingam K.M., Znati T. (2004). Anaysis of Wireless Sensor Networks for Habitat Monitoring. Wireless Sensor Networks.

[14] Britton M., Sacks L. The SECOAS Project: Development of a Self-Organising, Wireless Sensor Network for Environmental Monitoring.

[15] Tolle G., Polastre J., Szewczyk R., Culler D., Turner N., Tu K., Burgess S., Dawson T., Buonadonna P., Gay D. A macroscope in the redwoods.

[16] Werner-Allen G., Lorincz K., Ruiz M., Marcillo O., Johnson J., Lees J., Welsh M. (2006). Deploying a wireless sensor network on an active volcano. IEEE Internet Comput..

[17] Baggio A. Wireless sensor networks in precision agriculture.

[18] Delin K.A., Jackson S.P., Johnson D.W., Burleigh S.C., Woodrow R.R., McAuley J.M., Dohm J.M., Ip F., Ferré T.P.A., Rucker D.F. (2005). Environmental Studies with the Sensor Web: Principles and Practice. Sensors.

[19] Cardell-Oliver R., Kranz M., Smettem K., Mayer K. (2005). A Reactive Soil Moisture Sensor Network: Design and Field Evaluation. Int. J. Distrib. Sens. Netw..

[20] Martinez K., Riddoch A., Hart J., Ong R. (2006). A Sensor Network for Glaciers. Intelligent Spaces.

[21] Seders L.A., Shea C.A., Lemmon M.D., Maurice P.A., Talley J.W. (2007). LakeNet: An Integrated Sensor Network for Environmental Sensing in Lakes. Environ. Eng. Sci..

[22] Cano A., Lopez-Baeza E., Anon J.L., Reig C., Millan-Scheding C. Wireless Sensor Network for Soil Moisture Applications.

[23] O’Flynn B., Martinez R., Cleary J., Slater C., Regan F., Diamond D., Murphy H. SmartCoast: A Wireless Sensor Network for Water Quality Monitoring.

[24] Hakala I., Tikkakoski M., Kivela I. Wireless Sensor Network in Environmental Monitoring-Case Foxhouse.

[25] Barrenetxea G., Ingelrest F., Schaefer G., Vetterli M., Couach O., Parlange M. SensorScope: Out-of-the-Box Environmental Monitoring.

[26] Corke P., Wark T., Jurdak R., Hu W., Valencia P., Moore D. (2010). Environmental Wireless Sensor Networks. Proc. IEEE.

[27] Ayday C., Safak S. Application of Wireless Sensor Networks with GIS on the Soil Moisture Distribution Mapping.

[28] Antoine-Santoni T., Santucci J.-F., de Gentili E., Silvani X., Morandini F. (2009). Performance of a protected wireless sensor network in a fire. Analysis of fire spread and data transmission. Sensors.

[29] Choi S., Kim N., Cha H., Ha R. (2009). Micro sensor node for air pollutant monitoring: Hardware and software issues. Sensors.

[30] Kotamäki N., Thessler S., Koskiaho J., Hannukkala A.O., Huitu H., Huttula T., Havento J., Järvenpää M. (2009). Wireless *in-situ* Sensor Network for Agriculture and Water Monitoring on a River Basin Scale in Southern Finland: Evaluation from a Data User's Perspective. Sensors.

[31] Li M., Liu Y. (2009). Underground coal mine monitoring with wireless sensor networks. ACM Trans. Sens. Netw..

[32] Li X., Cheng X., Yan K., Gong P. (2010). A monitoring system for vegetable greenhouses based on a wireless sensor network. Sensors.

[33] Larios D.F., Barbancho J., Sevillano J.L., Rodríguez G., Molina F.J., Gasull V.G., Mora-Merchan J.M., León C. (2013). Five years of designing wireless sensor networks in the doñana biological reserve (Spain): an applications approach. Sensors.

[34] López J.A., Garcia-Sanchez A.-J., Soto F., Iborra A., Garcia-Sanchez F., Garcia-Haro J. (2010). Design and validation of a wireless sensor network architecture for precision horticulture applications. Precis. Agric..

[35] Yick J., Bharathidasan A., Pasternack G., Mukherjee B., Ghosal D. Optimizing placement of beacons and data loggers in a sensor network—A case study.

[36] Abbasi A.A., Younis M. (2007). A survey on clustering algorithms for wireless sensor networks. Comput. Commun..

[37] Younis M., Akkaya K. (2008). Strategies and techniques for node placement in wireless sensor networks: A survey. Ad Hoc Netw..

[38] Marks M. (2010). A Survey of Multi-Objective Deployment in Wireless Sensor Networks. J. Telecommun. Inf. Technol..

[39] Baronti P., Pillai P., Chook V. (2007). Wireless sensor networks: A survey on the state of the art and the 802.15. 4 and ZigBee standards. Comput. Commun..

[40] Cunningham K., Schrage L. (2013). LINGO: The Modeling Language and Optimizer.

[41] Kumar L., Skidmore A.K., Knowles E. (1997). Modelling topographic variation in solar radiation in a GIS Environment. Int. J. Geogr. Inf. Sci..

[42] Zimmermann N.E., Kienast F. (1999). Predictive mapping of alpine grasslands in Switzerland: Species *versus* community approach. J. Veg. Sci..

[43] Ball G.H., Hall D.J. (1965). Isodata, a Novel Method of Data Analysis and Pattern Classification.

[44] Kumarawadu P., Dechene D.J., Luccini M., Sauer A. Algorithms for Node Clustering in Wireless Sensor Networks: A Survey.

[45] Wark T., Hu W., Corke P., Hodge J., Keto A., Mackey B., Foley G., Sikka P., Brunig M. Springbrook: Challenges in developing a long-term, rainforest wireless sensor network.

[46] Jena R.K. (2010). Multi-Objective Node Placement Methodology for Wireless Sensor Network. Int. J. Comput. Appl. Spec. Issue MANETs.

[47] Molina G., Alba E., Talbi E.-G. (2008). Optimal Sensor Network Layout Using Multi-Objective Metaheuristics. J. Univers. Comput. Sci..

[48] Jourdan D.B., de Weck O.L. Layout optimization for a wireless sensor network using a multi-objective genetic algorithm.

[49] Jia J., Chen J., Chang G., Wen Y., Song J. (2009). Multi-objective optimization for coverage control in wireless sensor network with adjustable sensing radius. Comput. Math. Appl..

[50] Jena R.K., Mahanti P.K. Node Placement for Wireless Sensor Network Using Multi-objective.

[51] Álvarez N.G., Labrín B.C. Optimización de funciones a través de Optimización por Enjambre de Partículas y Algoritmos Genéticos.

[52] Yick J., Pasternack G., Mukherjee B., Ghosal D. (2006). Placement of network services in a sensor network. Int. J. Wirel. Mob. Comput..

[53] Gandham S.R., Dawande M., Prakash R., Venkatesan S. Energy efficient schemes for wireless sensor networks with multiple mobile base stations.

[54] Watson J.P., Greenberg H.J., Hart W.E. A multiple-objective analysis of sensor placement optimization in water networks.

[55] Hou Y.T., Sherali H.D., Midkiff S.F. (2005). On energy provisioning and relay node placement for wireless sensor networks. IEEE Trans. Wirel. Commun..

[56] Amaldi E., Capone A., Cesana M., Filippini I., Malucelli F. (2008). Optimization models and methods for planning wireless mesh networks. Comput. Netw..

[57] Vazirani V.V. (2003). Set Cover. Approximation Algorithms.

[58] Hillier F.S., Liberman G.J. (2010). Introducción a la Investigación de Operaciones.

